# Utilization of granular solidification during terrestrial locomotion of hatchling sea turtles

**DOI:** 10.1098/rsbl.2009.1041

**Published:** 2010-02-10

**Authors:** Nicole Mazouchova, Nick Gravish, Andrei Savu, Daniel I. Goldman

**Affiliations:** Georgia Institute of Technology, Atlanta, GA, USA

**Keywords:** Loggerhead sea turtle, biomechanics, locomotion, granular media, drag, limb

## Abstract

Biological terrestrial locomotion occurs on substrate materials with a range of rheological behaviour, which can affect limb-ground interaction, locomotor mode and performance. Surfaces like sand, a granular medium, can display solid or fluid-like behaviour in response to stress. Based on our previous experiments and models of a robot moving on granular media, we hypothesize that solidification properties of granular media allow organisms to achieve performance on sand comparable to that on hard ground. We test this hypothesis by performing a field study examining locomotor performance (average speed) of an animal that can both swim aquatically and move on land, the hatchling Loggerhead sea turtle (*Caretta caretta*). Hatchlings were challenged to traverse a trackway with two surface treatments: hard ground (sandpaper) and loosely packed sand. On hard ground, the claw use enables no-slip locomotion. Comparable performance on sand was achieved by creation of a solid region behind the flipper that prevents slipping. Yielding forces measured in laboratory drag experiments were sufficient to support the inertial forces at each step, consistent with our solidification hypothesis.

## 1. Introduction

Locomotion (Dickinson *et al*. 2000; Alexander 2003) on sand, a granular medium (Jaeger *et al*. 1996), is challenging because sand surfaces can flow during limb interaction and slipping can result, causing both instability and decreased locomotor performance (Lejeune *et al*. 1998). An important parameter that governs interaction of limbs with sand is the yield stress, defined as the force per unit area at which non-reversible material deformation occurs (Nedderman 1992). For a given geometry, for forces below *F*_yield_ the material behaves like an elastic solid, while above *F*_yield_ the material flows like a fluid dominated by friction between grains. This transition can have major effects on locomotor performance: our systematic studies of a bioinspired physical model (a robot SandBot (Li *et al*. 2009)) running on granular media revealed that, when limb kinematics were adjusted to use solidification features of the medium, the robot could achieve top speeds of approximately 50 per cent of those for hard ground. Slight changes in frequency and gait parameters lead to fluidization of the medium by the limb and catastrophic reductions in speed to 1 per cent of hard ground, predominantly because of decreased support forces and increased belly drag.

If organisms that move on sand exploit solidification properties of the medium, they could reap the benefits of anchored limb use during a step—these include reduction in dissipative energy loss associated with ground fluidization (Lejeune *et al*. 1998) and slipping. We hypothesize that organisms which move on sand can achieve performance comparable to that of non-yielding, rigid ground (which we assume provides the opportunity for maximal performance), using the solid properties of the granular media during stance. We test this hypothesis in an aquatic animal, the hatchling Loggerhead sea turtle (*Caretta caretta*), which must perform well on land to reach the ocean and avoid predation. Periodically, adult females travel to their natal beaches (Wyneken 1997), emerging from the sea to nest on land. After hatching, juveniles (hatchlings) climb from the nest and, travel distances up to several thousand body-lengths (BL) at speeds of several BL per second (N. Mazouchova 2008, personal observation). In the water they swim at average speeds of 5 BL s^−1^ using their aquatically adapted paddle-like flippers to generate hydrodynamic lift and thrust (Wyneken 1997). Although flippers are used on land for a tiny fraction of their lives (Hirth 1980), they enable excellent mobility over dune grass, rigid obstacles, and sand of varying compaction and moisture content.

Aerial and aquatic locomotive reaction forces (e.g. hydrodynamic thrust and lift) generated through interaction of wings and flippers can be analysed in detail through solution of the Navier–Stokes equations (Tritton 1989). Equivalent mechanisms have not yet been described and analysed at the same level for terrestrial locomotion on granular media (and other flowing terrain), in part, because comprehensive governing equations do not exist (Jaeger *et al*. 1996). However, empirical models can function well (Li *et al*. 2009; Maladen *et al*. 2009). In the SandBot experiments, a simple granular penetration model explained running speed versus limb frequency (Li *et al*. 2009). Here we use an empirical model of flipper interaction to support our biological observations, and demonstrate that on loose sand turtles' high performance can be achieved using solidification features of the granular medium.

## 2. Material and methods

**Figure 1.**
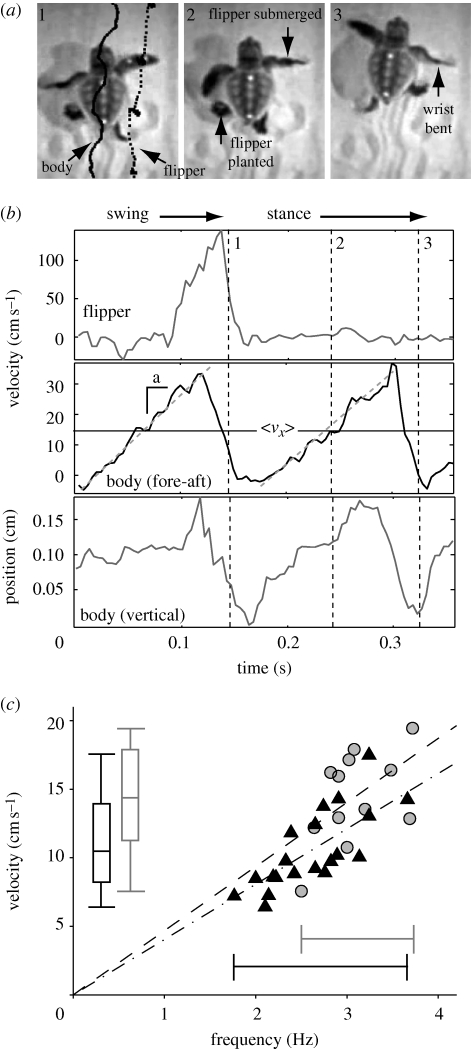
Sea turtle locomotion on sand. (*a*) Frame captures of tracked hatchlings on sand. (*b*) Flipper, body fore-aft velocity and vertical position over time; numbers correspond to frames in (*a*). (*c*) Velocity versus frequency for sand (black triangles) and hard ground (grey circles). Vertical bars show mean, s.d. and range of velocity while horizontal bars show range of frequency.

The study was conducted on Jekyll Island, GA, USA (electronic supplementary material, figure S1) in cooperation with the Georgia Sea Turtle Center. In 2008, there was a total of 166 nests, of which 10 nests were tested (*N*_sand_ = 18, *N*_hardground_ = 8, *n*_total_ = 26, with *N* as the number of animals) with turtle mass (19.5 ± 2.2 g), BL (6.9 ± 1.6 cm), flipper length (3.5 ± 0.9 cm) and flipper width (1.3 ± 0.2 cm). Hatchlings (electronic supplementary material, video S1) were collected during natural immersion and tests were performed in a mobile laboratory (electronic supplementary material, figure S2) containing a fluidized bed trackway (Li *et al*. 2009) filled with dry Jekyll Island sand. The bed allows preparation of the sand in a reproducible loosely packed state; air flow was cut-off during the experiments. A sandpaper board placed in the trackway was used to mimic hard ground. Two high-speed cameras (Sony Handycams, 240 fps under infrared light) recorded dorsal (figure 1*a*) and lateral images. Natural and removable markers (located on the carapace and the mid-point of the flipper) aided tracking of movement. Three runs per animal, with up to five animals, were recorded in a 2 h span. A run was considered successful if the animal took more than three steps, such that cycle average velocity returned to within 35 per cent of the velocity of the preceding step. Hatchlings were released at their collection location.

**Figure 2.**
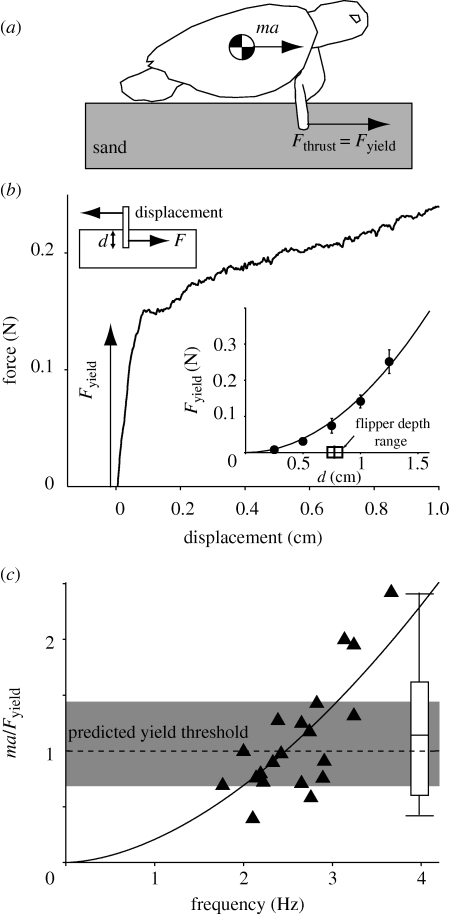
Model of locomotion on sand: (*a*) flipper ground reaction force *F*_thrust_ and inertial force *ma*. (*b*) Drag force versus displacement shows rapid rise in force (the yield force *F*_yield_) for small initial displacement. Inset: quadratic dependence of *F*_yield_ on insertion depth *d*. The bar shows range of measured flipper depths. (*c*) Normalized turtle inertial force (*ma*/*F*_yield_) versus limb frequency (fit curve is *ma*/*F*_yield_ = *cf^n^*; *c* = 0.21, *n* = 1.74, *r*^2^ = 0.65). Dashed line indicates predicted yielding threshold for a single flipper inserted to average measured turtle depth (grey region is yield for mean ± s.d. depth).

We performed laboratory measurements on a model flipper to estimate thrust forces. The model flipper consisted of a thin (1.45 mm) aluminum plate 3 cm long (comparable to flipper length) that was inserted into the Jekyll Island sand to the given penetration depth (*d* = 0.25–1.25 cm) and dragged at 0.05 cm s^−1^ over a distance of 5 cm; as in other experiments (Maladen *et al*. 2009), drag force was independent of speed up to 20 cm s^−1^. Calibrated strain gauges mounted to the model flipper provided force measurements during drag. Displacement was controlled by a stepper motor and lead-screw. Force data were sampled at 1 kHz. Yield force of the media was determined from the *y*-intercept of a linear fit to the drag force after motion of the plate began (figure 2*b*).

## 3. Results

Despite the different contact mechanics associated with sand and sandpaper, forward velocity of the body (close to centre of mass) *v*_*x*_ versus time was similar on both substrates. At each step, *v*_*x*_ increased from zero to a maximum then dropped rapidly to zero again (figure 1*b*). Average speed on sand was reduced by 28 per cent (better than SandBot performance loss) relative to hard ground, but maximal speeds were the same in both treatments. Turtles employed a diagonal gait (Wyneken 1997) with average stance duty factors of 0.66 ± 0.05. During each stride, the body was lifted off the ground by an average of 2.2 ± 0.9 mm, and then touched down at the end of the cycle (figure 1*b*). Average *v*_*x*_ increased linearly with stride frequency *f* (in hertz) as 〈*v*_*x*_〉 = *sf* (figure 1*c*) with similar stride length, *s* = 4.0 ± 1.9 cm on sand and *s* = 4.7 ± 2.9 cm on hard ground; *s* was significantly different from 0 for all treatments (*p* < 0.0001) and the slope of the regressions were not statistically different (*p* > 0.05). Average inertial force (*ma*) on sand increased with frequency (figure 2*c*). Limb kinematic measurements revealed that the angular extent of the shoulder excursion did not depend on the treatment (sand: 111 ± 17°, sandpaper: 114 ± 6°; *p* > 0.05) in accord with the derived stride length.

On sand, at touchdown, pressure owing to the thin (approx. 2 mm wide) edge of the flipper exceeded the vertical yield stress and it penetrated into the sand. The limb (shoulder) rotated as the flipper penetrated until the flipper was perpendicular to the surface. The rotation served to lift (figure 1*b*) the body (see discussion of model below and in the electronic supplementary material, SI). During thrust, the portion of the flipper in the sand (approx. 3 cm long and 0.76 cm deep on average) at first remained perpendicular to the direction of motion (relative flipper surface-forward velocity angle during mid-stance was 99.4 ± 16.9°) and later was adducted, as both the wrist and shoulder rotated and the body moved forward and upward (electronic supplementary material, video S2). On sandpaper, a claw at the wrist engaged irregularities and propelled the animal forward; during thrust the shoulder rotated towards the body and the wrist did not bend keeping the limb fully extended. A tracked marker on the mid-point of the flipper (figure 1*a*,*b*) demonstrated that limb slip was minimal on both substrates (net displacement of >1 flipper-width in only 2.6% of steps on sandpaper and 5.6% on sand) during forward movement, consistent with equivalent stride lengths.

## 4. Discussion

Our results imply that speeds on sand and hard ground are similar, because for both treatments limbs do not slip during locomotion, stride length is constant and 〈*v*_*x*_〉 = *sf*. On hard ground, no-slip is maintained by a claw engaging irregularities. On sand, entirely different mechanics account for no-slip: in successful runs, material behind the flipper did not move during the thrust phase, supporting the hypothesis that the turtle advances via solidification of the material behind it.

Forward movement of the body on sand without slipping of the flipper requires that net thrust forces *F* _thrust_ remain below the yield force of the granular medium, *F*_thrust_ < *F*_yield_. We assume that the mechanics of the large front flipper (maintaining surface normal vector parallel to *v*_x_) produces the dominant contribution to *F*_thrust_. Observation of the smaller hind limbs indicate that at initiation of stance, the foot remains plantar and above the surface during the entire step, presumably contributing to lifting the body and less to thrust (force measurements in a different turtle species (Wyneken 1997) shows evidence that they are used for lifting although no force data exists for Loggerheads). Since the animal lifts at each stride using both hindlimbs and forelimbs (figure 2*a*), we assume that the plastron is not in contact during the thrust phase and thus body drag is not significant.

Therefore, *F*_thrust_ needs to generate only the inertial forces (mass × acceleration; *ma*) required to accelerate the animal from rest to its maximum velocity (figures 1*b* and 2*a*). As plastron elevation removes drag during the stride, locomotion is governed by *F*_thrust_ = *ma*. We estimate average inertial forces from linear fits of the body velocity during the acceleration phase of the movement (figures 1*b* and 2*b*). Since *v*_peak_ = 2.88 〈*v*_*x*_〉 (*r*^2^ = 0.88), and 〈*v*_*x*_〉 is proportional to *f*, we expect average inertial forces (*ma* ∝ *v*_peak_*f*) during a step to increase as *f*^2^ (see electronic supplementary material). The data are consistent with this prediction (figure 2*c*).

We estimated ground reaction forces from the drag of a model flipper. Drag force on a plate (figure 2*b*) increased sharply within the first millimetre of displacement. We identify the force at the end of this sharp increase as *F*_yield_, since it is generated in a short distance and no large-scale flow of material occurs. *F*_yield_ increases as the square of the penetration depth (figure 2*b*) and linearly with plate width (Wieghardt 1975). The existence of *F*_yield_ thus provides a possible mechanism for solidification and generation of thrust forces on sand without slipping, by using the solid properties of the media. If *ma* < *F*_yield_ (or *ma/F*_yield_ < 1), material solidifies during the power stroke.

Choosing *F*_yield_ at the average measured flipper insertion depth, *d* = 0.76 ± 0.13 cm, reveals that the majority of the derived fore-aft acceleration data satisfy the criterion *ma*/*F*_yield_ < 1 (figure 2*c*) and thus indicates that the material can remain solid with the use of a single flipper. Only at the greatest accelerations does the model predict slip. We do not observe limb slip in these runs, and speculate that, at these large accelerations, the hind limb contributes by friction from its plantar surface (we estimate that if the hind flipper supports half the turtle's weight on sand, with a measured surface friction coefficient of µ = 0.6, the thrust/*F*_yield_ from friction, µ mg/(2*F*_yield_) ≈ 0.6 would be sufficient to account for the largest observed inertial forces). Force platform data are needed to determine the individual contributions to thrust from forelimbs and hindlimbs. In addition, we hypothesize that *F*_yield_ can be increased if limb rotation during entry (which could enhance normal loading and material compaction) is considered; further physics experiments are needed to test this hypothesis.

Our model reveals that a major challenge for rapid locomotion on sand is the balance between high speed, which requires large inertial forces, and the potential for failure through fluidization, which can occur when inertial forces (which increase sensitively like *f* ^2^) exceed *F*_yield_. In the SandBot experiments, failures through fluidization could be induced by the reduction of *F*_yield_ through changes in material compaction (Li *et al*. 2009). Since *F*_yield_ depends on many factors, including particle properties and hill angle, it may be ecologically important to examine performance (and possible locomotor failures) as a function of substrate properties like beach topography (inclines) or sand type (e.g. through renourishment (Steinitz *et al*. 1998)).

In conclusion, high-performance locomotion on yielding substrates such as sand can be achieved using the solid-like response governed by the yield stress. Further biological studies and physical models of turtles (and other organisms) are required to determine if and how organisms control limb movements to remain below the yield stress on granular media. More broadly, to discover principles of passive and active nervous and mechanical system control (Nishikawa *et al*. 2007), as well as to understand energetic costs (Lejeune *et al*. 1998) in locomotion on and within realistic terrain, will require advances in theory and experimental characterization of complex media. Otherwise we must continue to rely on empirical force laws specific to particular geometries, kinematics and granular media.
